# Annotations

**Published:** 1898-02-12

**Authors:** 


### Annotations.

### The Queen's Speech.

Several subjects were touched upon in the Queen's Speech which are of special interest to the medical profession. The plague in India is referred to, and it is stated that "although the mortality is less alarming than it was at this time last year, it is still such as to cause anxiety." It is also said that no efforts will be spared to limit its extent and to mitigate its effects, and we may, perhaps, express a hope that these efforts will include a full recognition of the necessity for placing all sanitary measures under the control of a proper medical staff. Amid the proposals "having for their object to secure increased strength and efficiency in the army, and for amending the present conditions of military service," we hope that special attention will be paid to the feeding of the soldiers. There seems no reason why young soldiers should be docked of their pay for the purchase of provisions any more than young sailors are. Then there is that relic of barbarism, "flogging in the army," which is generally imagined to have been done away with, but is still retained in military prisons. Let us hope it will be put an end to. Lastly, there comes the great question, will the Government have the good sense to satisfy the medical profession in regard to the status of the officers of the army medical service? for on this must depend much of the efficiency of the army as a fighting force. That is the point. As a show force the army can grub on without a proper medical service, but as a fighting force it must have doctors. It is with much satisfaction that we see that "a measure for the amendment of the vaccination law will be recommended" to the earnest attention of Parliament. This is a distinct promise, and we hope that the measure to be introduced will be such as to settle this question for long to come. Among a bundle of measures to be brought forward, "in case the time . . . . should permit," is one "for the constitution of a Teaching University for London," and one for preventing the adulteration of drugs and food. This bundle of "innocents" looks suspiciously as if it were meant for "slaughter"; but we hope that at least the Teaching University will survive. It would be a scandalous thing if a Government with such a majority should neglect the claims of London in so important a matter.

### The Prince of Wales's Hospital Fund.

The managers of every hospital will be grateful to the Council of the Prince of Wales's Hospital Fund for the economy in the expenditure, which amounts to the small sum of £1 12s. 6d. per cent. on the sum raised. This is the first occasion on which so large a sum has been raised for the hospitals on the voluntary system at so inappreciable a cost. The amount of office work entailed by the proper checking of every letter and promise of money, owing to the great number of small amounts contributed, is a striking testimony to the care and ability of the management of the honorary secretaries and assistant secretaries. The lion's share of the work has fallen upon Sir Savile Crossley and Mr. J. G. Craggs, and it gives us infinite pleasure to be able to announce that Her Majesty the Queen has recognised these services by presenting each of them with the Jubilee medal. The excellent system of control and the strict enforcement of economy which has been practised will be better appreciated when it is understood that the expenses include salaries, office expenses, and the gross sum charged for advertisements, although much of the latter item has been reduced by contributions from the newspaper proprietors who have generously given considerable donations to the Fund. Had these donation been deducted from the charges for advertisements, the total cost of the collections would probably have been reduced to about 1 per cent. It is desirable that these facts should be widely known, because they not only afford matter for sincere congratulation, but they must tend to impress everybody who desires to support the hospitals with a feeling that they cannot do better, if they have no special knowledge of hospitals, than send their gifts to the Prince of Wales's Fund.

### Motherhood and Militarism.

So great are the evils connected with the employment of married women in factories that many would be inclined to exclude them altogether from that sort of work. Besides the injury done by working in mills during the later months of pregnancy, the absence of mothers from home involves an early recourse to artificial feeding in the rearing of infants, who moreover are apt to be neglected in many ways when they are handed over to strangers for the greater part of the day. Even on the economic side there is but little to be said in favour of the practice. The wife's earnings are but a supplementary source of income, and there is much reason for believing that the effect of married women's work is rather to pull down the general wages than to increase the family earnings. On every hand, therefore, except so far as concerns interference with personal liberty, the argument is entirely in favour of restricting the factory work of married women. In Germany, however, although the Government is paternal to an extent which we can hardly understand here, and the people would perfectly willingly obey any edict restricting women's work, a very curious objection is said to stand in the way. Everything in Germany has to be looked at from a military point of view, and when it becomes a question of deciding such a matter as this it has to be considered, not as it concerns the mothers, or the wages, or the manufacturers, or the comfort of the home. The soldiers are a first necessity—the work of the women of Germany is to produce them, and for this purpose it becomes a matter of State policy to encourage matrimony. But the Germans are a careful people, and what costs too much money they go without. They count the cost of all their pleasures, and even matrimony they will forego if it seems too expensive. To the careful youths of the Fatherland the prospect of a wage-earning wife is sweet; but it is feared that if the wife were kept out of the factory, and if the prospective husband saw that he would have to support the partner of his joys, he would just do without her. And, then, what about the children, and the soldiers, and the Empire? So German mothers must needs go on working in the factories, lest perchance the German maidens should not get married, and should thus fail in their duty to the army. Such are the exigencies of a military system.

